# Reemergence of African Swine Fever in Zimbabwe, 2015

**DOI:** 10.3201/eid2305.161195

**Published:** 2017-05

**Authors:** Juanita van Heerden, Kerstin Malan, Biko M. Gadaga, Reverend M. Spargo

**Affiliations:** Agricultural Research Council–Onderstepoort Veterinary Institute, Onderstepoort, South Africa (J. van Heerden, K. Malan);; Central Veterinary Laboratory, Harare, Zimbabwe (B.M. Gadaga); Veterinary Services, Harare (R.M. Spargo)

**Keywords:** African swine fever, genotype II, reemergence, Zimbabwe, Africa, southern Africa, eastern Africa, swine, pigs, ticks, pig farms, epidemiology, transmission, viruses

## Abstract

Zimbabwe is the only country in southern Africa with no reported African swine fever (ASF) outbreaks during 1993–2014. However, the 2015 discovery of genotype II ASF virus in Zimbabwe indicates the reemergence of ASF in this country and suggests that this viral genotype may be spreading through eastern and southern Africa.

In many countries in Africa, pig farm production is restrained by outbreaks of African swine fever (ASF), a highly contagious and rapidly spreading disease that causes high rates of death among infected pigs and for which there is no vaccine (*1*). In southern Africa, the epidemiology of the causative agent, ASF virus (ASFV), is further complicated by the presence of sylvatic and domestic transmission cycles. The sylvatic cycle has been implicated in most outbreaks reported near national parks where infected warthogs (*Phacochoerus africanus*) and soft tick (*Ornithodoros* spp*.*) vectors are present (*1*). The virus can be horizontally transmitted between pigs via the uncontrolled movement of virus-contaminated pig products and the feeding of swill containing infectious pig meat; transmission is also facilitated by the lack of adequate biosecurity measures (*2*).

Genotyping is used to determine relationships between ASFV strains (*3*). ASF became endemic in Eurasia after the spread of a genotype II ASFV from eastern Africa to Georgia in 2007 (*4*,*5*). The virus has continued to spread in Eurasia, and in 2014, it was detected in Lithuania and Poland, putting the European Union at risk (*6*). In eastern and southern Africa, genotype II ASFV has been described in Tanzania, Mauritius, and Mozambique (*7*). Zimbabwe had outbreaks caused by ASFV genotype I in 1990 and 1992 and by genotype VIII in 1961. No further outbreaks were reported in Zimbabwe until July 2015, when ASF was detected in domestic pigs in Mashonaland Central Province in northern Zimbabwe, where it was confined to the area along the border with Mozambique. The virus spread locally because of the salvage slaughter of infected pigs, the selling of infected pig meat at discounted prices, the movement of pigs between villages, and the inappropriate disposal of infectious carcasses.

During the 2015 outbreak in Zimbabwe, pigs with ASF exhibited fever (temperature 41°–42°C), dullness, anorexia, and swaying gait. Light-colored pigs also showed reddening of the skin on the ears and abdomen, but redness was not evident on the indigenous black breed of pigs. Postmortem examinations revealed bloody discharge from the anus and nostrils; edema of the lungs; and hemorrhages in the mesenteric lymph nodes, kidney, and heart.

We pooled the organs from 3 infected pigs from 3 separate villages in the outbreak area and extracted DNA. We amplified the variable 3′ end of the B646L (p72) gene by using 2 oligonucleotide primers, p72-U and p72-D (*3*). To amplify the entire E183L gene (p54), we used primers described by Oviedo et al. (*8*), and to amplify the tetramer amino acid repeats within the hypervariable central variable region of the B602L gene, we used primers described by Gallardo et al. (*5*). Our results showed that sequences for all ASFV isolates from this outbreak and those for isolates previously collected in eastern Europe and eastern Africa were 100% homologous over the p72, p54, and central variable region gene-coding regions. The p72 sequences clustered in genotype II (Figure).

**Figure:**
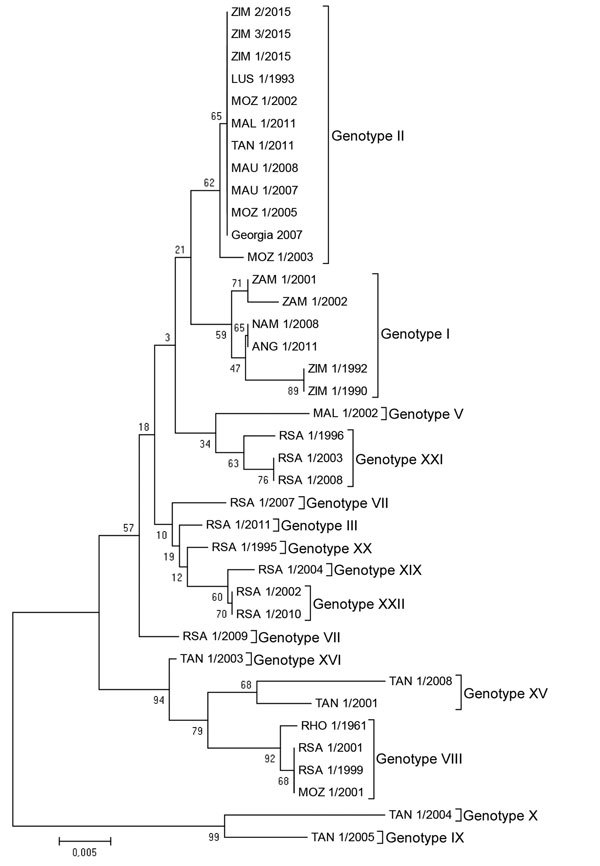
Neighbor-joining phylogenetic tree based on the partial B646L (p72) gene sequences of African swine fever virus (ASFV) isolates from a 2015 outbreak in Zimbabwe. The outbreak strains (ZIM/1/15, ZIM/2/15, and ZIM/3/15 [GenBank accession nos. KX090921–KX090923]) grouped with genotype II ASFV strains isolated in Mozambique (MOZ), Tanzania (TAN), Malawi (MAL), Mauritius (MAU), and Georgia, sharing 100% nucleotide identity with those strains. Phylogeny was inferred after 1,000 bootstrap replications; values at nodes indicate the percentage of bootstrap support. Scale bar indicates nucleotide substitutions per site.

During the 2015 ASFV outbreak in Zimbabwe, a total of 3,427 pigs were at risk for infection in the affected area. Of those, 2,836 (≈83%) became infected, and all infected pigs died. The 591 pigs that did not become infected had been confined in pens and did not have exposure to infected pigs or their products. A follow-up study is under way in the region to genetically characterize the viruses in this outbreak, focusing on the p54, p30 and, central variable region genes.

All villages affected during the 2015 outbreak in Zimbabwe were along the northern border with Mozambique, where genotype II has been found before. It is essential that more of the ASFVs circulating in eastern and southern Africa be sequenced so that their relatedness can be determined. This knowledge will enable the establishment of an epidemiologic link between outbreaks in the region and underscore the need for adequate quarantine measures to prevent ASF from becoming endemic in southern and eastern Africa.
